# The complete chloroplast genome of *Salix lindleyana* (salicaceae), a plateau plant species

**DOI:** 10.1080/23802359.2023.2246675

**Published:** 2023-08-21

**Authors:** Nan Xu, Xin Du, Xiu-Xing Zhang, Hai-Ling Yang

**Affiliations:** The Tree and Ornamental Plant Breeding and Biotechnology Laboratory of National Forestry and Grassland Administration; Institute of Tree Development and Genome Editing, College of Biological Sciences and Biotechnology, Beijing Forestry University, Beijing, China

**Keywords:** *Salix lindleyana*, chloroplast genome, phylogenetic analysis

## Abstract

*Salix lindleyana* Wallich ex Andersson 1851 is a species of genus *Salix* which mainly grows on mountains above 3000 m at sea level in Qinghai–Tibetan Plateau (including the Himalayas and Hengduan Mountains). To determine its phylogenetic position within *Salix*, we reconstructed *S. lindleyana* complete chloroplast (cp) genome sequence by de novo assembly using whole-genome sequencing data. The completed chloroplast genome was 155,304 bp, with a total GC content of 36.7%. It had a very typical tetrad structure, including a large single-copy (LSC) region of 84,539 bp, a small single-copy (SSC) region of 16,161 bp, and two inverted repeats (IR) regions of 27,302 bp. A total of 132 functional genes were distributed in the chloroplast genome, including 87 protein-coding genes, 37 tRNA genes, and 8 rRNA genes. Phylogenetic analysis showed that *S. lindleyana* was clustered with *Salix dasyclados* Wimmer 1849 and *Salix variegata* Franchet 1887. The complete chloroplast genome of *S. lindleyana* provides potential genetic resources for further phylogenetic studies.

## Introduction

*Salix lindleyana* Wallich ex Andersson 1851, a member of the genus *Salix*, is a cushion-like shrub with a prostrate and rooted main trunk, and only a few centimeters high. It is more common in the wet rock crevices at altitudes over 3000 m at sea level in Qinghai–Tibetan Plateau (including Himalayas and Hengduan Mountains). *S. lindleyana* grows in an environment with large temperature difference between day and night, high ultraviolet radiation intensity, and strong wind. To adapt to the complex and changeable geographical environment of the plateau, the plant shape and the genome sequence of *S. lindleyana* have been continuously evolved, which makes it of important ecological value and high research value. However, the genome of *S. lindleyana* has not been sequenced or assembled. In this study, the complete chloroplast of *S. lindleyana* was first assembled and annotated to explore it‘s genomic structure, and a phylogenetic tree of *S. lindleyana* and other willows was also constructed to understand their evolutionary relationships.

## Material and methods

**Figure 1.**
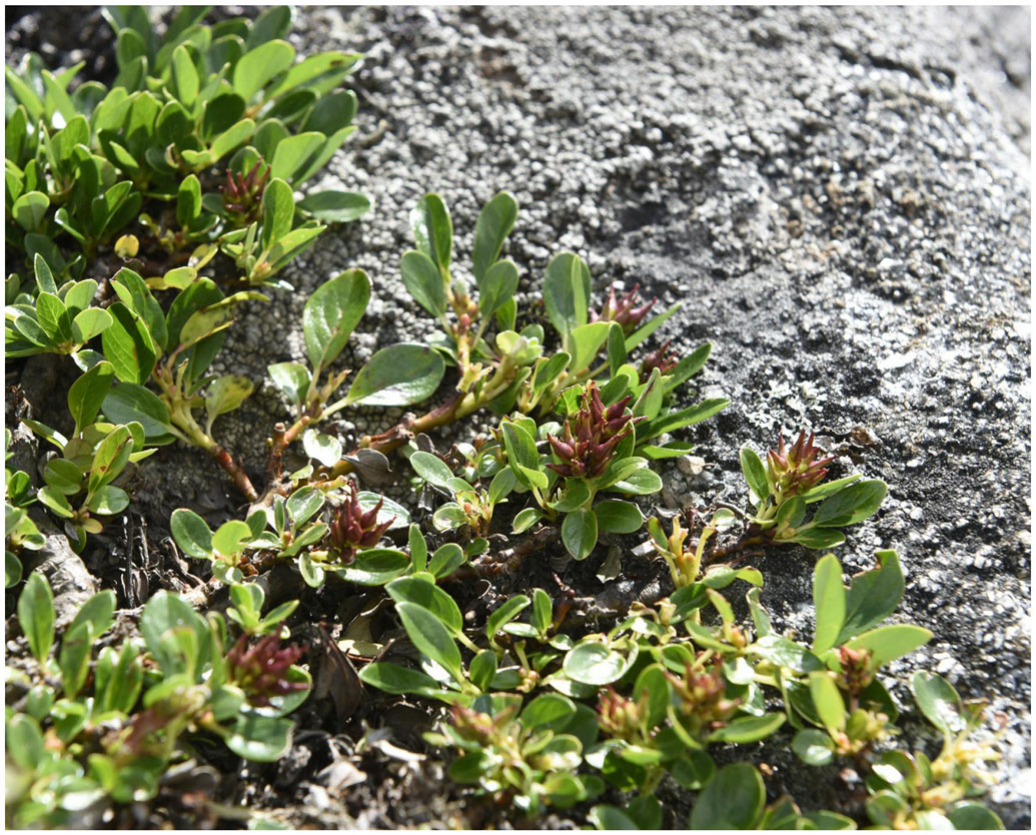
An individual of *Salix lindleyana* (species reference map was taken by Xin Du and Xiu-Xing zhangin Kangding City, Ganzi Tibetan Autonomous Prefecture, Sichuan Province, China (29°54′18″N, 102°0′7″E). *Salix lindleyana* is a kind of cushion shrub, which stem prostrate and rooting. Leaves obovate-oblong or obovate-lanceolate, bright green above, glabrous, midrib distinctly concave. Inflorescences ovate, with only a few flowers per inflorescence, born at the tip of the current branch. Bracts broadly ovate, apex rounded, pale purplish-red.

We collected leaf samples from Kangding City, Ganzi Tibetan Autonomous Prefecture, Sichuan Province, China, at an altitude of 4010 m, with specific coordinates of 29°54′18″N, 102°0′7″E (Figure 1). This article was licensed under the Regulations of Strategy of Sichuan Province on Biodiversity Conservation and approved by the Beijing Forestry University (Beijing, China). A specimen was deposited at the herbarium of Beijing Forestry University (http://bjfc.bjfu.edu.cn, Zhi-Xiang Zhang, zxzhang@bjfu.edu.cn) under the voucher number BJFC00099886. Total genomic DNA was extracted by the CTAB method (Doyle and Doyle JL 1987), and sequenced using Illumina high-throughput sequencing platform NovaSeq 6000. We used the default parameters of GetOrganelle v1.7.5 (Jin et al. 2020) software for plant chloroplast assembly of DNA sequencing data to obtain a complete circular plant chloroplast genome (default parameters: -R 15-k 21, 45, 65, 85, 105-F embplant_pt). CPGAVAS2 (Shi et al. 2019) software was used to annotate the chloroplast genome with reference to the chloroplast genome of *Arabidopsis thaliana* (Sato et al. 1999) and CPGView (Liu et al. 2023) software was used to visualize the chloroplast genome. Each annotation error in the chloroplast genome is manually corrected using the Apollo (Lewis et al. 2002) software. To clarify the accuracy of the assembly, we further mapped our clean reads back to the assembled chloroplast (cp hereafter) genome to assess the depth of coverage (Figure S1).

To evaluate phylogenetic position of *S. lindleyana*, another 31 *Salix* complete chloroplast genomes were downloaded from GenBank database. The chloroplast genome of *Populus trichocarpa* (EF489041) (Tuskan et al. 2006) was used as outgroup. Sequences were aligned by MAFFT v7.310 (Katoh et al. 2019) with default parameters, and the phylogenetic tree was constructed by maximum likelihood method using Phyml v3.3 (Guindon et al. 2010) with the GTR + I + G model and 1,000 rapid bootstraps. The amino acid substitution model was calculated by modelgenerator (Keane TM et al. 2006). The completed chloroplast genome of *S. lindleyana* was submitted to GenBank with accession number OM892926.

## Results

**Figure 2.**
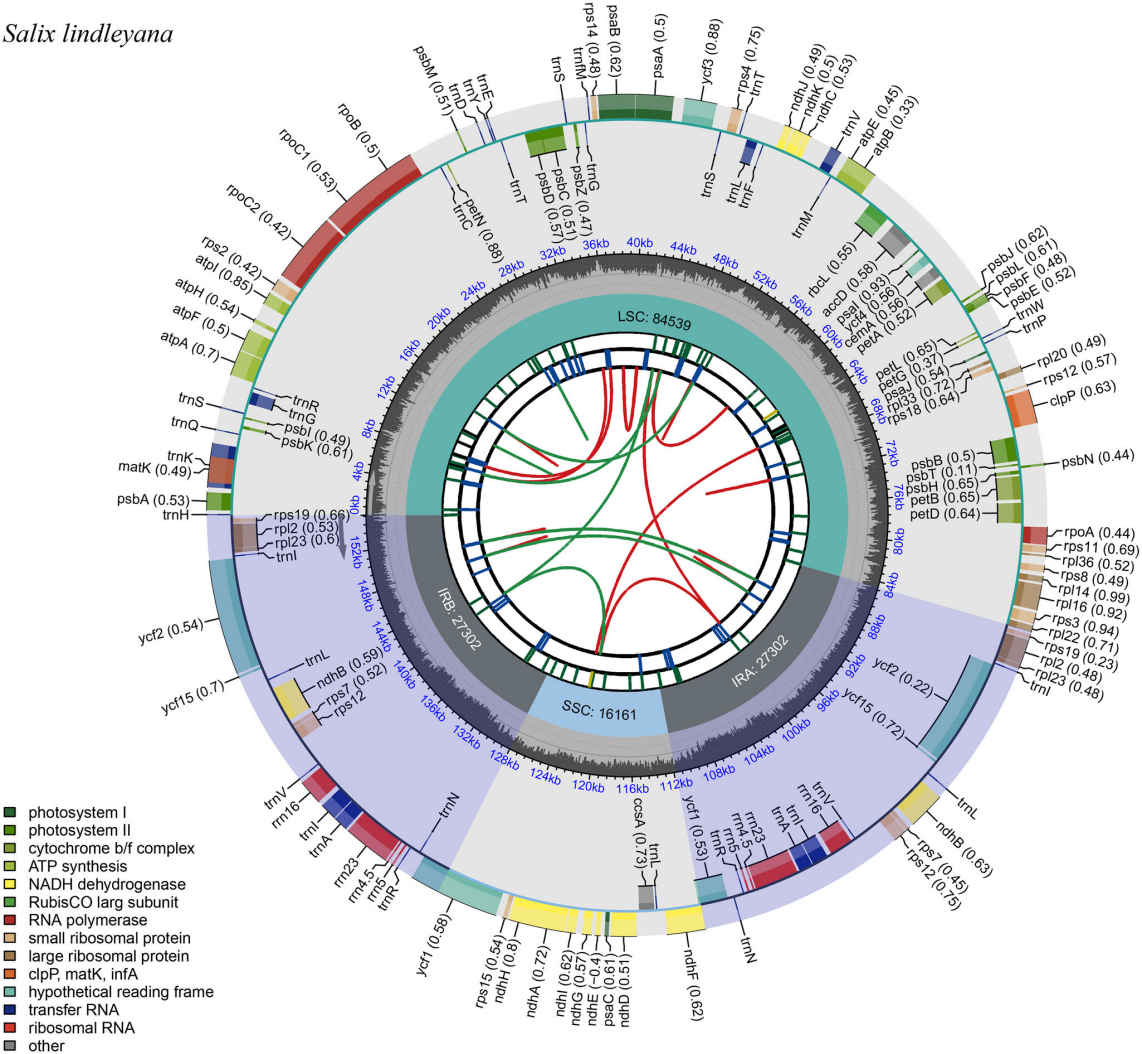
Schematic representation of the plastome features of *Salix lindleyana*. From the center outward, the first track shows the dispersed repeats. The dispersed repeats consist of direct (D) and palindromic (P) repeats, connected with red and green arcs. The second track shows the long tandem repeats as short blue bars. The third track shows the short tandem repeats or microsatellite sequences as short bars with different colors. The colors, the type of repeat they represent, and the description of the repeat types are as follows. Black: c (complex repeat); green: p1 (repeat unit size = 1); yellow: p2 (repeat unit size = 2); purple: p3 (repeat unit size = 3); blue: p4 (repeat unit size = 4); orange: p5 (repeat unit size = 5); red: p6 (repeat unit size = 6). the small single-copy (SSC), inverted repeat (IRa and IRb), and large single-copy (LSC) regions are shown on the fourth track. The GC content along the genome is plotted on the fifth track. The base frequency at each site along the genome will be shown between the fourth and fifth tracks. The genes are shown on the sixth track. The optional codon usage bias is displayed in the parenthesis after the gene name. Genes are color-coded by their functional classification. The transcription directions for the inner and outer genes are clockwise and anticlockwise, respectively. The functional classification of the genes is shown in the bottom left corner.

The total length of the chloroplast genome of *S. lindleyana* (OM892926) was 155,304 bp, of which the large single-copy (LSC) region was 84,539 bp, the small single-copy (SSC) region was 16,161 bp, and the inverted repeat (IR) region was 27,302 bp (Figure 2). The circular map of the chloroplast genome was generated using CPGview (Liu et al. 2023). A total of 132 functional genes were identified after annotation including 877 protein-coding genes, 37 tRNA-coding genes, and 8 rRNA-coding genes. Among these genes, 19 genes contained introns, including 8 tRNA (*trn*K-UUU, *trn*G-GCC, *trn*L-UAA, *trn*V-UAC, two *trn*I-GAU and two *trn*A-UGC) and 11 protein-coding genes (*ndh*A, *rpo*C1, *atp*F, *pet*B, *pet*D, *rpl*16, *ycf*3*,* two *rpl*2 and two *ndh*B). Seven tRNA genes (*trn*I-CAU*, trn*L-CAA*, trn*V-GAC*, trn*I-GAU*, trn*A-UGC*, trn*R-ACG and *trn*N-GUU), four rRNA genes (*rrn*16S, *rrn*23S, *rrn*4.5S and *rrn*5S) and nine protein-coding genes (*rps*19, *rp*l2, *rpl*23, *ycf*2, *ycf*15*, ndh*B*, rps*7*, rps*12 *and ycf*1 were duplicated and located on the IR regions. Fourteen genes (*atp*F, *rpo*C1, *pet*B, *pet*D, *rpl*16, *rpl*2, *ndh*B, *ndh*A, *trn*K-UUU, *trn*G-GCC, *trn*L-UAA, *trn*V-UAC, *trn*I-GAU, *trn*A-UGC) contained one intron (Figure S2), whereas *rps*12 and *ycf*3 contained two introns, *rps*12 was also a trans-spliced gene (Figure S3). The overall GC content of the chloroplast genome was 36.7%, and the IR regions (42.0%) had higher GC content than the SSC (31.0%) and the LSC regions (34.4%).

**Figure 3.**
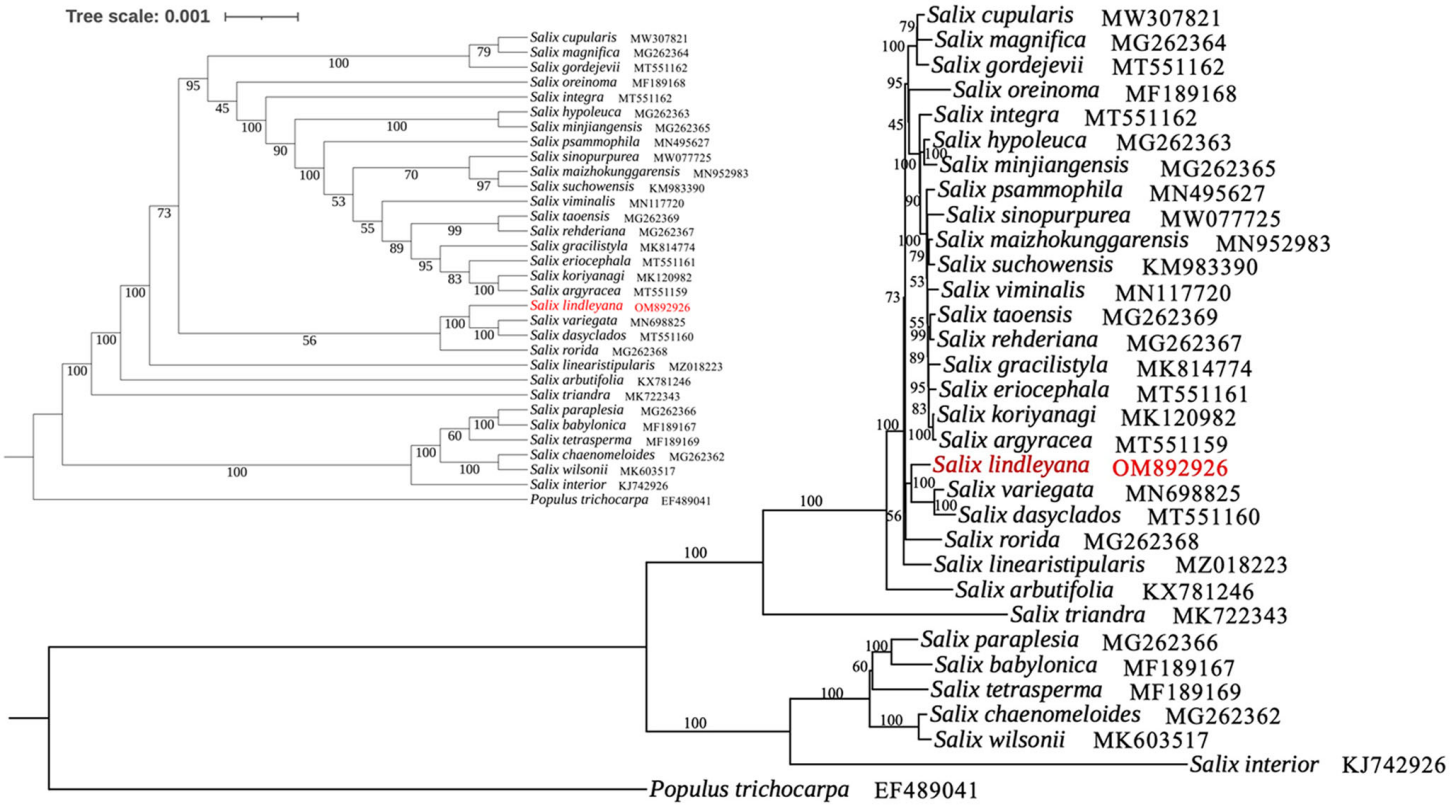
Phylogenetic tree of 32*Salix* species was established with maximum likelihood using the complete chloroplast genome. The cladogram tree was placed in the upper left corner. Number on each node indicated bootstrap support value. The following sequences were used: *Salix cupularis* (MW307821; Li et al. 2021), *Salix magnifica* (MG262364; Liu et al. 2020), *Salix gordejevii* (MW562004; Wei and Li 2021), *Salix oreinoma* (MF189168; Huang et al. 2017), *Salix integra* (MT551162; Zhou et al. 2021), *Salix hypoleuca* (MG262363; unpublished), *Salix minjiangensis* (MG262365; unpublished), *Salix psammophila* (MN495627; Lu et al. 2019), *Salix sinopurpurea* (MW077725; Guo et al. 2021), *Salix maizhokunggarensis* (MN952983; Ma et al. 2020), *Salix suchowensis* (MG262365; Wu 2016), *Salix viminalis* (MN117720; Hu et al. 2019), *Salix taoensis* (MG262369; unpublished), *Salix rehderiana* (NC_037427; unpublished), *Salix gracilistyla* (MK814774; Xi et al. 2019), *Salix eriocephala* (MT551161; unpublished), *Salix koriyanagi* (MK120982; Kim et al. 2019), *Salix argyracea* (MT551159; unpublished), *Salix variegate* (MN698825; Chen 2020), *Salix dasyclados* (MT551160; unpublished), *Salix rorida* (MG262368; unpublished), *Salix linearistipularis* (MZ018223; ren et al. 2021), *Salix arbutifolia* (KX781246; unpublished), *Salix triandra* (MK722343; Wu et al. 2019(a)), *Salix paraplesia* (MG262366; unpublished), *Salix babylonica* (MF189167; Huang et al. 2017), *Salix tetrasperma* (MF189169; Huang et al. 2017), *Salix chaenomeloides* (MG262362; unpublished), *Salix wilsonii* (MK603517; Wu et al. 2019(b)), *Salix interior* (KJ742926; Huang et al. 2014).

The relationship among 32 *Salix* could be well revealed by the phylogenetic tree which constructed with chloroplast genome datas (Figure 3). The results suggested that *S. lindleyana* as a sister group to *Salix dasyclados* Wimmer and *Salix variegata* Franchet with high bootstrap support.

## Discussion and conclusions

The chloroplast genome of *S. lindleyana* was the second report of *Sect. Lindleyanae Schneid* which further complement the genome information of plateau *Salix*. The complete chloroplast genome of *S. lindleyana* provides potential genetic resources for further evolutionary and genomic studies on *Salix*.

Chloroplast genomes with high conservation have been widely used in species identification and phylogenetic analysis (Szymon et al. 2016). Wagner’s research suggested that *Salix* had lower plastid variation than other angiosperms, making it unsuitable for phylogenetic reconstruction (Wagner et al. 2021). However, Zhou’s study on the whole genome phylogeny and classification of chloroplasts in five *Salix* species found that *ycf*1, *psa*I, *ycf*2-2, *rpo*C2, *rpl*22, *atp*F and *ndh*F genes were under positive selective in 21 *Salix* species. *Rps7* is the most variable region in 21 *Salix* chloroplast genomes and can be used as a molecular marker for species identification (Zhou et al. 2021). These results indicated that chloroplast genome can provide reference for phylogenetic and evolutionary studies of *Salix*.

He et al. (2021) use drestriction‐site associated DNA (RAD) sequencing data to reconstruct the phylogeny and spatiotemporal evolution. *Salix lindleyana* was clustered in subclades I of the Hengduan Mountains clade and was a sister group to *S. cff. flabellaris* Andersson 1860, which was consistent with monophyly of taxonomic sections. Alpine dwarf willow branches (*S. lindleyana*, *S. cff. flabellaris*, *S. oreinoma* C. K. Schneid. in Sargent 1916, and *S. opsimantha* C. K. Schneid. in Sargent 1916) differentiated at 6.93-8.1 Ma, and *S. oreinoma* clustered in subclade II, showing adaptability to the high-altitude niche.

This study constructed the phylogenetic tree with chloroplast genome dates to reveal the relationship among 32 *Salix.* Phylogenetic analysis of chloroplast genome showed that shrub willows and tree willows were separated in phylogenetic tree. The shrub willows were divided into two distinct branches. *S. lindleyana*, *Salix variegata*, and *Salix dasyclados* form an independent clade. This was very similar to the clustering results of subclasses I and II of the mountain clade of HDM. *S. oreinoma* clustering in other branches was consistent with the differentiation of alpine dwarf willow branches in He’s study. The phylogenetic results in this study were partially consistent with the nuclear genome. However, due to the limited chloroplast genome data of the members of *Salix* genus, it was hard to fully display the phylogenetic relationship of the plastid genome, and the comparison with nuclear genome evolution was still one-sided.

## Supplementary Material

Supplemental Material

Supplemental Material

Supplemental Material

## Data Availability

The genome sequence data that support the findings of this study are openly available in the GenBank of NCBI at https://www.ncbi.nlm.nih.gov under the accession number OM892926. The associated BioProject, SRA, and Bio-Sample numbers are PRJNA835694, SRR19116050, and SAMN28108208 respectively.

## References

[CIT0001] Chen J. 2020. Characterization of the complete chloroplast genome of *Salix variegata* (Salicaceae). Mitochondrial DNA Part B Resour. 125(1):196–197. doi: 10.1080/23802359.2019.1698989. PMC7748512 33366484

[CIT0002] Doyle JJ, Doyle JL. 1987. A rapid DNA isolation procedure for small quantities of fresh leaf tissue. Phytochem Bulletin. 19(1980):11–15.

[CIT0003] Guindon S, Dufayard J-F, Lefort V, Anisimova M, Hordijk W, Gascuel O. 2010. New algorithms and methods to estimate maximum-likelihood phylogenies: assessing the performance of PhyML 3.0. Syst Biol. 59(3):307–321. doi: 10.1093/sysbio/syq010. 20525638

[CIT0004] Guo F, Liu K, Wang Y, Li E, Zhan Z, Zhang Z. 2021. Complete chloroplast genome sequence of *Salix sinopurpurea* (Salicaceae). Mitochondrial DNA B Resour. 116(3):718–719. doi: 10.1080/23802359.2020.1858726. PMC7954483 33763559

[CIT0005] He L, Wagner ND, Hörandl E. 2021. Restriction-site associated DNA sequencing data reveal a radiation of willow species (Salix L., Salicaceae) in the Hengduan Mountains and adjacent areas. J Syst Evol. 59(1):44–57. doi: 10.1111/jse.12593.

[CIT0006] Hu H-L, Lin H, Chen X-H, Liu Y-Q, Qin L. 2019. The complete chloroplast genome sequence of *Salix viminalis* (Salicaceae). Mitochondrial DNA Part B Resour. 184(2):3035–3036. doi: 10.1080/23802359.2019.1666041. PMC7706889 33365844

[CIT0007] Huang DI, Hefer CA, Kolosova N, Douglas CJ, Cronk QCB. 2014. Whole plastome sequencing reveals deep plastid divergence and cytonuclear discordance between closely related balsam poplars, *Populus balsamifera* and *P. trichocarpa* (Salicaceae). New Phytol. 204(3):693–703. doi: 10.1111/nph.12956. 25078531

[CIT0008] Huang Y, Wang J, Yang Y, Fan C, Chen J. 2017. Phylogenomic Analysis and Dynamic Evolution of Chloroplast Genomes in *Salicaceae*. Front Plant Sci. 8:1050. doi: 10.3389/fpls.2017.01050. 28676809 PMC5476734

[CIT0009] Zhou J, Jiao Z, Guo J, et al. 2021. Complete chloroplast genome sequencing of five Salix species and its application in the phylogeny and taxonomy of the genus, Mitochondrial DNA Part B. 6 (8): 2348–2352. 34345693 10.1080/23802359.2021.1950055 PMC8284152

[CIT0010] Jin J-J, Yu W-B, Yang J-B, Song Y, dePamphilis CW, Yi T-S, Li D-Z. 2020. GetOrganelle: a fast and versatile toolkit for accurate de novo assembly of organelle genomes. Genome Biol. 21(1):241. doi: 10.1186/s13059-020-02154-5. 32912315 PMC7488116

[CIT0011] Katoh K, Rozewicki J, Yamada KD. 2019. MAFFT online service: multiple sequence alignment, interactive sequence choice and visualization. Brief Bioinform. 20(4):1160–1166. doi: 10.1093/bib/bbx108. 28968734 PMC6781576

[CIT0012] Keane TM, Creevey CJ, Pentony MM, Naughton TJ, Mclnerney JO. 2006. Assessment of methods for amino acid matrix selection and their use on empirical data shows that ad hoc assumptions for choice of matrix are not justified. BMC Evol Biol. 6(1):1–17. doi: 10.1186/1471-2148-6-29. 16563161 PMC1435933

[CIT0013] Kim J, Kim Y, Park J. 2019. Complete chloroplast genome sequence of the *Salix koriyanagi* Kimura ex Goerz (Salicaceae). Mitochondrial DNA Part B: res. 4(1):549–550. doi: 10.1080/23802359.2018.1553521.

[CIT0014] Lewis SE, Searle SMJ, Harris N, Gibson M, Lyer V, Richter J, Wiel C, Bayraktaroglu L, Birney E, Crosby MA, et al. 2002. Apollo: a sequence annotation editor. Genome Biol. 3(12):RESEARCH0082. doi: 10.1186/gb-2002-3-12-research0082. 12537571 PMC151184

[CIT0015] Li J, Zhuo Z, Xu D, Yang H, Zhu T. 2021. The complete chloroplast genome of *Salix cupularis* Rehder, a sand binder in alpine hillslope. China. Mitochondrial DNA Part B Resour. 6(9):2519–2520. doi: 10.1080/23802359.2021.1959435. PMC8330782 34377814

[CIT0016] Liu J, Liu S, Wang R, Guan S, Qu J. 2020. Characterization of the complete chloroplast genome of *Salix magnifica*, a vulnerable species endemic to China. Mitochondrial DNA Part B Resour. 25(3):2269–2270. doi: 10.1080/23802359.2019.1710602. PMC7510628 33367003

[CIT0017] Liu S, Ni Y, Li J, Zhang X, Yang H, Chen H, Liu C. 2023. CPGView: apackage for visualizing detailed chloroplast genome structures. Mol Ecol Resour. 23(3):694–704. doi: 10.1111/1755-0998.13729. 36587992

[CIT0018] Lu D, Hao L, Huang H, Zhang G. 2019. The complete chloroplast genome of *Salix psamaphila*, a desert shrub in northwest China. Mitochondrial DNA Part B Resour. 4(2):3432–3433. doi: 10.1080/23802359.2019.1675485. PMC7707305 33366026

[CIT0019] Ma R, Zhou M, Wang Y, Zhu G, Li J, Feng Q, Miao N. 2020. Characterization of the complete chloroplast genome of *Salix maizhokunggarensis* (Salicaceae). Mitochondrial DNA Part B Resour. 35(1):1054–1055. doi: 10.1080/23802359.2020.1721374. PMC7748764 33366871

[CIT0020] Sato S, Nakamura Y, Kaneko T, et al. 1999. Complete structure of the chloroplast genome of Arabidopsis thaliana. DNA Res. 6(5):283–290. doi: 10.1093/dnares/6.5.283. 10574454

[CIT0021] Shi L, Chen H, Jiang M, Wang L, Wu X, Huang L, Liu C. 2019. CPGAVAS2, an integrated plastome sequence annotator and analyzer. Nucleic Acids Res. 47(W1):W65–W73. doi: 10.1093/nar/gkz345. 31066451 PMC6602467

[CIT0022] Szymon AO, Ewelina Ł, Tomasz K, et al. 2016. Chloroplasts: state of research and practical applications of plastome sequencing. Planta. 244:517–527. 27259501 10.1007/s00425-016-2551-1 PMC4983300

[CIT0023] Tuskan GA, DiFazio S, Jansson S, Bohlmann J, Grigoriev I, Hellsten U, Putnam N, Ralph S, Rombauts S, Salamov A, et al. 2006. The genome of black cottonwood, *Populus trichocarpa* (Torr. & Gray). Science. 313(5793):1596–1604. 15doi: 10.1126/science.1128691. 16973872

[CIT0024] Wagner ND, Volf M, Hörandl E. 2021. Highly diverse Shrub Willows (Salix L.) Share Highly Similar Plastomes. Front Plant Sci. 12:662715. doi: 10.3389/fpls.2021.662715. 34539686 PMC8448165

[CIT0035] Wall. ex Andersson. Salix lindleyana Wall. ex Andersson. 1851. Kongl. Svenska Vetenskapsakad. Handl. 1850: 499.

[CIT0036] Wall. ex Andersson. Salix lindleyana latifolia Andersson. Kongl. 1851. Kongl. Vetensk. Acad. Handl. 1850: 497

[CIT0025] Wei Y, Li X. 2021. Characterization of the complete chloroplast genome of *Salix gordejevii* (Salicaceae). Mitochondrial DNA Part B Resour. 6(9):2510–2511. doi: 10.1080/23802359.2021.1959438. PMC8330744 34377810

[CIT0026] Wu Z. 2016. The whole chloroplast genome of shrub willows (*Salix suchowensis*). Mitochondrial DNA Part A DNA Mapp Seq Anal. 27(3):2153–2154. 10.3109/19401736.2014.982602 25418623

[CIT0027] Wu D, Wang Y, Zhang L, Dou L, Gao L. 2019a. The complete chloroplast genome and phylogenetic analysis of *Salix triandra* from China. Mitochondrial DNA Part B Resour. 154(2):3571–3572. doi: 10.1080/23802359.2019.1674743. PMC7707385 33366090

[CIT0028] Wu D, Wang Y, Zhang L. 2019b. The complete chloroplast genome sequence of an economic plant *Salix wilsonii*. Mitochondrial DNA Part B Resour. 154(2):3560–3562. doi: 10.1080/23802359.2019.1668311. PMC7707237 33366085

[CIT0029] Xi H, Park J, Kim Y. 2019. The complete chloroplast genome sequence of rose-gold pussy willow, *Salix gracilistyla*Miq. (Salicaceae). Mitochondrial DNA Part B Resour. 104(2):2118–2120. doi: 10.1080/23802359.2019.1623115. PMC7687643 33365434

[CIT0030] Zhou J, Jiao Z, Guo J, Wang B s, Zheng J. 2021. Complete chloroplast genome sequencing of five *Salix* species and its application in the phylogeny and taxonomy of the genus. Mitochondrial DNA Part B Resour. 156(8):2348–2352. doi: 10.1080/23802359.2021.1950055. PMC8284152 34345693

