# Supplementary figures and images for: The complete chloroplast genome of Salix lindleyana (salicaceae), a plateau plant species
Source: Mitochondrial DNA B Resour. 2023 Aug 21;8(8):877–81. doi: 10.1080/23802359.2023.2246675 (PMC10443960; doi:10.1080/23802359.2023.2246675)

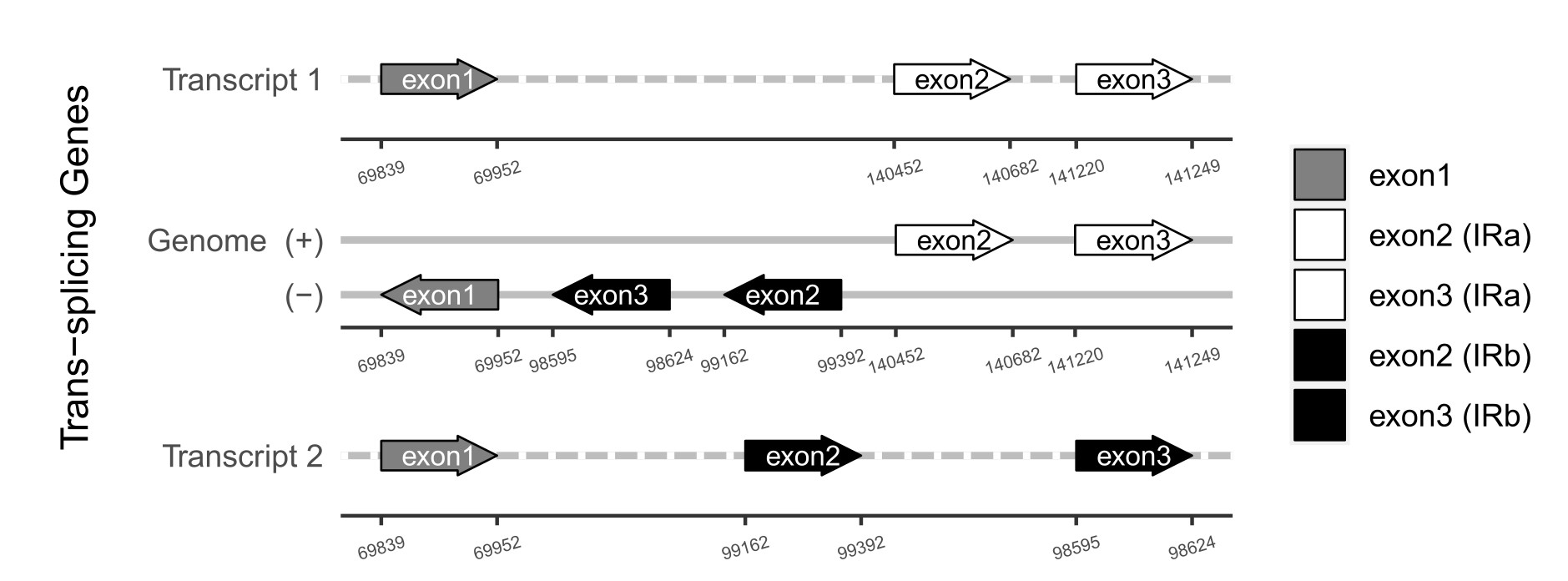

Supplement: Supplemental Material [file TMDN_A_2246675_SM0530.jpg]

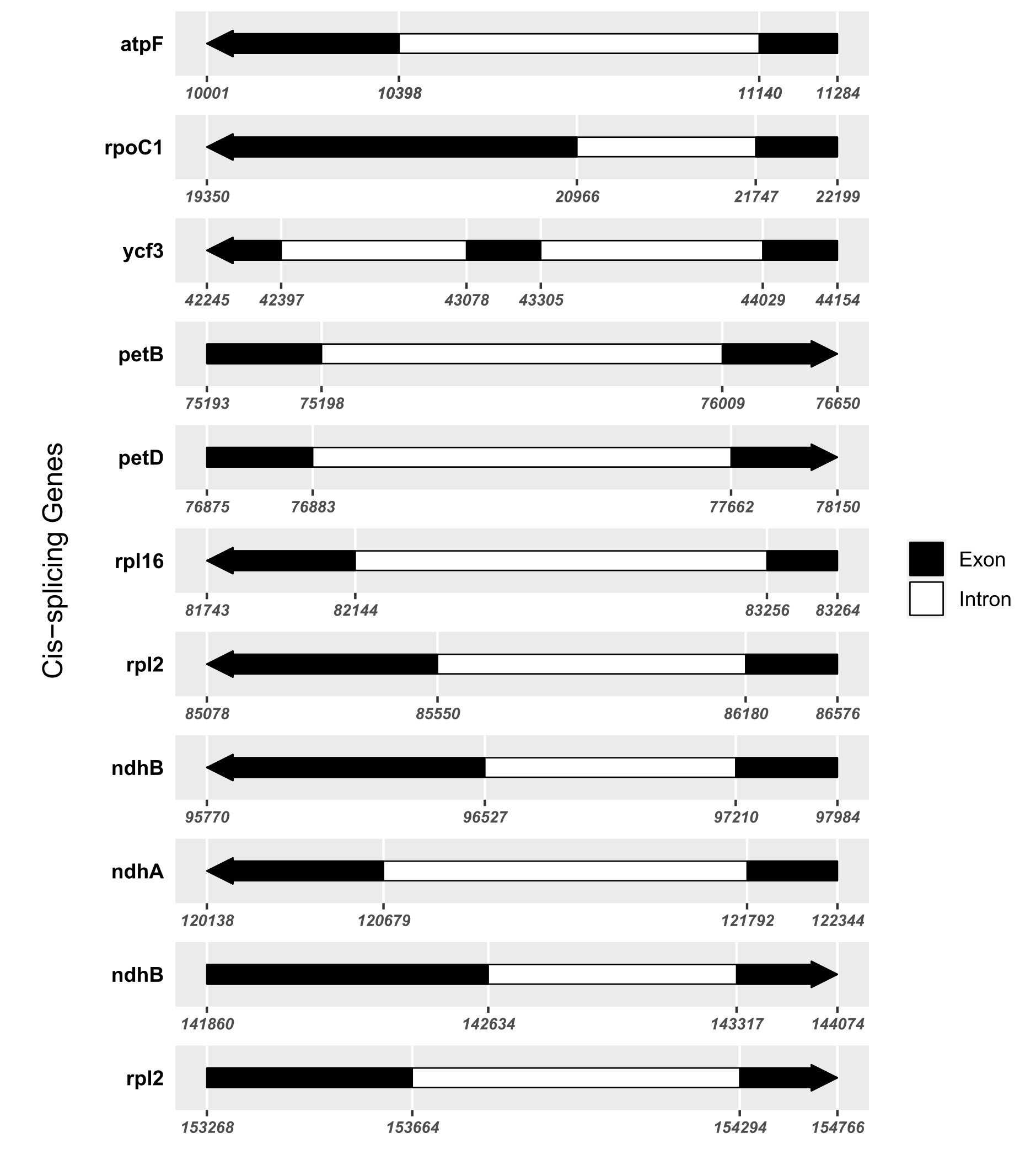

Supplement: Supplemental Material [file TMDN_A_2246675_SM0529.jpg]

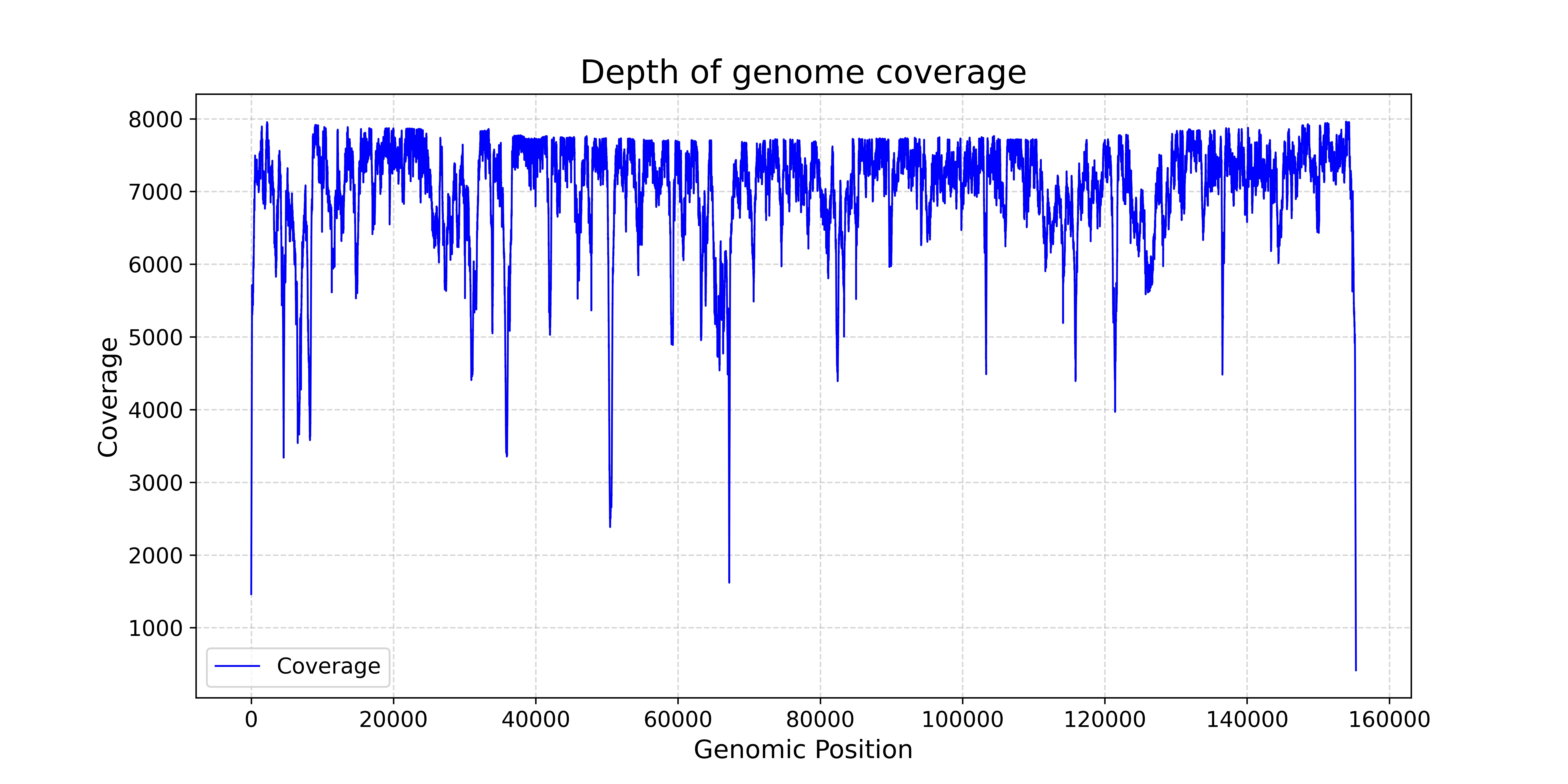

Supplement: Supplemental Material [file TMDN_A_2246675_SM0528.jpg]
